# Socio-economic factors associated with periodontal conditions among Brazilian elderly people - Multilevel analysis of the SBSP-15 study

**DOI:** 10.1371/journal.pone.0206730

**Published:** 2018-11-07

**Authors:** Rafael Aiello Bomfim, Antonio Carlos Frias, Claudio Mendes Pannuti, Celso Zilbovicius, Antonio Carlos Pereira

**Affiliations:** 1 School of Dentistry, Department of Community Health, Federal University of Mato Grosso do Sul (UFMS), Campo Grande, Mato Grosso do Sul, Brazil; 2 School of Dentistry, Department of Community Health, University of São Paulo (USP), São Paulo, São Paulo, Brazil; 3 School of Dentistry, Department of Periodontology, University of São Paulo (USP), São Paulo, São Paulo, Brazil; 4 School of Dentistry, Department of Community Health, State University of Campinas (UNICAMP), Piracicaba, São Paulo, Brazil; University Lyon 1 Faculty of Dental Medicine, FRANCE

## Abstract

The objective of the present study was to analyze individual, contextual and social support factors associated with periodontal condition among 2332 dentate elderly Brazilian participants from the São Paulo State Oral Health Survey 2015 (SBSP-15). Methods: This study used the database compiled by the SBSP-15, conducted from January to December in 2015. The associations were made by relative risk (RR), with Multilevel Poisson Regressions, among individual, contextual and social support variables, and had periodontal diseases as outcomes. Results: The mean age of elders was 70.13 years (SD 5.67). The risk factors for all outcomes of periodontal diseases were male gender and self-perceived color of non-white skin. Regarding social support, feeling unhappy was a risk factor for the presence of shallow periodontal pockets (3–5mm) RR 1.43(CI 95% 1.10–1.86). The coverage of the Family Health Strategy (FHS) was a protective factor for gingival bleeding RR = 0.7(CI 95% 0.44–0.99) and calculus RR = 0.75(CI 95% 0.60–0.95), and a risk factor for the number of lost sextants (sextants with only one tooth or without any teeth) RR = 1.12(CI 95% 1.00–1.28). Living in municipalities with more than 90% fluoridation coverage was a protective factor for the number of lost sextants RR = 0.89(CI 95% 0.78–0.99). Conclusions: The study showed evidence that individual (gender and self-perceived skin color), contextual (coverage of the family health strategy and water fluoridation) and social support factors (feeling unhappy) are associated with the clinical outcomes of periodontal diseases in Brazilian elders. This reinforces the need for transdisciplinary actions in the FHS, stimulating work together and intersectoral collaboration between FHS and NASF (Family Health Support Center).

## Introduction

The proportion of the Brazilian elderly population has increased due to the epidemiological transition process that has been occurring in Brazil[1]. Changes in the age structure of the general population affect the demand for oral health services, including periodontal dental services, and call for greater information on prevalence and incidence to be gathered, so that healthcare planning can effectively provide for the elderly population and best meet its needs[2].

Oral disease is considered a major public health problem in several countries, considering that poor oral health can cause not only systemic effects, but also a negative impact on quality of life[3]. The presence and severity of these effects were associated with socially disadvantaged populations, thus suggesting a correlation between periodontal disease and social inequalities[4]. With this in mind, it is of paramount importance to identify the treatment needs of elderly individuals and the associated factors influencing periodontal diseases. This knowledge would allow dental structures to be preserved and would prevent against the worsening of systemic diseases, related to periodontal status, such as diabetes mellitus[5].

Kassebaum et al (2014)[6], using the WHO classification, estimated a global average (GA) of severe periodontitis (SP) of 11.2%, which increased gradually with age, and with large variation around countries. The prevalence and incidence of SP in Brazil is higher than the GA, so policy makers should be aware of Brazilian periodontal status because life expectancy is increasing and tooth loss is decreasing around the world, this encourages research across countries on periodontal diseases, especially among this age group and the possible factors that might be associated.

Some studies have reported the influence of social support on the outcome of oral diseases in the elderly Japanese, such as avoiding social isolation and living in areas with a larger network of friends. Among the outcomes is the likelihood of having 20 or more teeth in their mouth[7]. Social support is a vast field, and has fostered a broad field of research[8]. Furthermore, it can greatly contribute to identifying the public policies for the healthcare sector.

Within this perspective, national surveys are important tools to help identify the health conditions of the population[9], and thus ensure that decisions on national policy will be properly grounded. With aging, individual, contextual, and social support characteristics may increase the potential for developing diseases. The coverage of the family health strategy (FHS) was a significant factor in protecting against periodontal disease[10], dental treatment needs[11], and self-perception of treatment need[12] in the Brazilian elderly population. However, to the best of our knowledge, no studies have been conducted on the influence of fluoridation on the public water supply and social support on periodontal treatment needs among elderly Brazilians.

Therefore, the objective of the present study was to evaluate the potential influence of contextual, individual, and social support variables on the periodontal conditions of Brazilian elderly participants in the São Paulo Oral Health 2015 (SBSP-15) survey, an epidemiological survey carried out in the state of São Paulo.

## Materials and methods

### Study design

This study was developed with secondary data derived from the SBSP-15 epidemiological survey and from contextual characteristics of cities in the state of São Paulo, evaluated in this survey. The SBSP-15 was a statewide epidemiological cross-sectional study on oral health, which evaluated different diseases in different age groups conducted from January to December in 2015. The study aimed at surveying the oral health conditions of the population from the state of São Paulo in the age groups of adolescents (15–19 years), adults (35–44 years) and elderly people (65 years and over), from 6 macroregions representing the entire state of São Paulo. For this purpose, 178 municipalities were drawn, and the state capital (Primary Sampling Units–PSUs) was included. In the second stage, 390 census sectors (Secondary Sampling Units–SSUs) (2 sectors for 177 municipalities and 36 sectors for the city of São Paulo) were drawn. The sampling plan was determined in two stages, with a probability proportional to size (PPS) of the population[13,14].

The information in the present study was collected by individual household interviews with a structured questionnaire. The research work was carried out exclusively in households of selected municipalities drawn from the six macroregions of the state of São Paulo. The exhaustion technique with minimum sample size was used for each PSU. ^13, 14^ Training and calibration process of dental teams (dentists and assistants) were conducted with kappa (>0.76). The data were collected by a clinical examination under natural light, with an oral mirror and a spherical tip probe, conducted by teams consisting of a dentist and an assistant[14].

The SBSP epidemiological survey evaluated the periodontal conditions of the elderly participants, aged over 65 years (n = 5951), using the Community Periodontal Index (CPITN), which is recommended by the World Health Organization (WHO)[15]. The need for clinical periodontal treatment was evaluated based on data for the presence of sextants with dental calculus, shallow periodontal pockets (3–5 mm) and deep periodontal pockets (≥ 6 mm). The index consisted of independently recording the presence or absence of five indicators in each sextant examined:

### Outcomes

1-Gingival Bleeding after probing; 2- Dental Calculus; 3—Shallow periodontal pockets (3 mm to 5 mm);4—Deep periodontal pockets (6 mm or more) and 5 –Lost sextants (Sextants with only one tooth or without any teeth were excluded, and consider in this category outcome)

The elderly participants could be included in more than one category if they had at least one sextant with the presence of more than one indicator, for example, a sextant with deep periodontal pocket and with dental calculus. If no index teeth were present in the sextant, all the remaining teeth in that sextant were examined, except for the distal surface of the third molars. The presence of two or more teeth with no indication of tooth extraction was considered a prerequisite for inclusion of the sextant in the periodontal examination.

The correlation between periodontal conditions and both individual and contextual factors for the year 2015 was evaluated only in elderly people who were not edentulous (n = 2332). The following contextual factors were analyzed: Municipal Human Development Index (MHDI)[16], Gini Index[16], population coverage by the FHS oral health teams[17] and fluoridation. The use of fluoridation was assessed according to information from the national sanitation system (SNIS -2015)[18], and was stratified into three levels: 1—absence of fluoridation or below 70% of population coverage; 2—above 70% and below 90% of fluoridation coverage; and 3—above 90% of fluoridation coverage in the respective city. The MHDI is a geometric measure assessing dimensions of income, education and longevity. It ranges from 0 to 1, where the higher the value, the better the social conditions. The MHDI was pooled as follows: less than or equal to 0.7 and greater than 0.7, according to the United Nations Development Program (UNDP) Atlas[16]. The Gini Index is a statistical measure assessing income inequality and ranges between 0 and 1, where 0 corresponds to absolute equality and 1, to absolute inequality[16]. The Gini index was grouped according to distribution in tertiles: lowest tertile, medium tertile and upper tertile.

The coverage of the population estimated by the FHS Oral Health Team program for the municipalities selected by SBSP-15 was used as an indicator of the availability and ease of access to services covered by the oral health teams, and was grouped as: less than 25%, between 25% and 50%, and more than 50%.

The FHS contextual variable corresponds to the average monthly number of primary oral health teams for every 1000 families (about 3000 individuals) in relation to the total population of the municipality in the year analyzed. The Ministry of Health[17] establishes 50% of oral health coverage as a parameter, but we chose to stratify into less than 25%, 25–50%, and more than 50%, especially because many cities have less than 25% coverage. Table 1 shows the description of the individual variables analyzed. Data on individual factors were collected through individual interviews conducted with a structured questionnaire at the homes of the interviewees. The information was collected as independent variables, as follows: gender, skin color, schooling and family income. Education was assessed by estimating the number of years of education completed without failing any grades, and was a continuous variable. Self-perceived skin color was classified as white and non-white (black, brown and other), and monthly family income was grouped as: up to $ 1500.00 and above $ 1500.00. All the variables analyzed are described in Table 1.

**Table 1:** Independent variables according to analysis level and SDH category.

level	Classification	Variable	Description
1st level-	Intermediate SDH	Gender	Sex of individual (male/female)
Individual		Self-declare skin color	Self-declare skin color (white or non-white)
		Schooling	Completed years of study, continuous variable
		Age Group	Age of elderly (65to 69 years and >70)
		Monthly income	Total income of all residents (above R$1500 and under)
	Social Capital	Cooperation	very/relative cooperation; indifferent and improbably
		Security	very/relative secure; indifferent and improbably
		Happiness	very/relative happy; indifferent and unhappiness
2nd level-	Structural SDH	2010 HMDI	summarized measure of basic living(<0,7;0,7–0,79;>0,8)
Contextual		Gini coefficient	measure of deviation of distribution of wealth
	Intermediate SDH	Fluoride water supply	condition of public water supply regarding water fluoridation
		Oral Health Teams	Population coverage by Family Health Strategy

SDH–Social Determinants of Health

### Measuring social capital variables

The variables of social capital and/or social support were related to cooperation, security and happiness, stratified into three levels each, and was extracted from Social Capital- Integrated questionnaire (SC-IQ)[19]. Cooperation was stratified into cooperative, indifferent and non-cooperative and the question was: “If there was a water supply problem in this community, how likely is it that people will cooperate to try to solve the problem?”[19], Security, into feeling safe, indifferent and insecure, and the question was: “In general, how safe from crime and violence do you feel when you are alone at home?”[19] and Happiness, into feeling happy, indifferent or unhappy toward life and the question was: “In general, how happy do you consider yourself to be?”[19].

### Ethical aspects

Prior to initiating the present study, the research protocol was evaluated and approved by the Research Ethics Committee of FOP UNICAMP—Final Opinion Number: 1211025 (2015), CAEE no. 46788215.9.0000.5418, according to resolution 466/12 of the National Health Council, for research on human beings. A free and informed consent term was applied and signed by each person examined in the study.

### Data analysis

Correlations between conditions and both individual periodontal and contextual factors were estimated by relative risk (RR), with a significance level of 5% and a 95% confidence interval (95% CI). RR values ​​were obtained using multilevel Poisson regression models with mixed effects models (random and fixed), and with Stata software, version 14.0 (StataCorp., College Station, TX, USA). RR estimated the associations between the dependent variables and the individual, contextual and social capital factors with a 5% significance level and a 95% CI.

## Results

The total sample participating in SBSP-15 was 2332 dentate elders. The mean number of teeth was 15.11 (SD± 8.16), and 77% of elders had at least one lost sextant, 59.39% had at least one sextant with dental calculus and 8.4% had deep Periodontitis (periodontal pockets > 6mm).

Regarding contextual factors, 77.48% lived in municipalities with a high MHDI; 42.12% lived in municipalities with higher income inequality; 42.20% had over 50% FHS oral health coverage; and 74% of the municipalities had over 90% fluoridation of the public water supply, as observed in Table 2. Regarding social support, 75.87% considered themselves very / relatively cooperative, 61.41%, very / relatively safe, and 87.84%, very / relatively happy.

**Table 2:** Descriptive and contextual variables of Brazilians elderly (n = 2332).

Variable		%	IC95%
**Contextual**			
**Gini**			
Lower	28.50	26.69	30.39
Middle	29.38	27.55	31.28
Upper (greatest inequality)	42.12	40.09	44.14
**IDHM**			
≤ 0,699	1.80	1.39	2.52
0,70–0,79	77.48	75.72	79.14
>0,79	20.72	19.03	22.35
**Family health Strategy- oral teams**			
<25%	25.66	23.92	27.5
25–50%	32.14	30.25	34.08
>50%	42.20	40.18	44.23
**water fluoridation**			
0–70%	10.23	9.1	11.54
70–90%	14.86	10.64	20.74
>90%	74.9	86.89	89.53
**Individual**			
**Gender**			
Male	43.90	41.88	45.94
Female	56.10	54.06	58.12
**Self-declare skin color**			
White	68.39	66.44	70.26
Non White	31.61	29.73	33.55
**Income**			
< R$1500,00	45.82	43.79	47.87
> R$1500,00	54.18	52.12	56.21
**Age group**			
65–69 years	52.20	50.16	54.25
>70 years	47.8	45.74	49.84
**Periodontal Outcomes**			
Gingival bleeding	43.48	41.48	45.5
Calculus	59.39	57.38	61.36
Shallow pocket	26.30	22.60	30.20
Deep pocket	8.40	6.53	11.24
Lost sextants	77.06	70.22	84.32

Analysis of individual, contextual and social support characteristics revealed that individual characteristics were associated with all the outcome variables analyzed. Female gender was an overall protective factor, and reduced the chance of gingival bleeding by 24%, dental calculus by 28%, shallow pockets by 26% and deep pockets by 35%. Conversely, according to the adjusted RR coefficient, being a woman was a risk factor for the number of lost sextants, which increased by 10%. Self-reported non-white skin color was a risk factor for all outcome variables analyzed, with the exception of the number of lost sextants. Schooling was a protective factor for the number of lost sextants, where the higher the schooling, the lower the number of sextants lost. Income was a protective factor for the number of lost sextants only according to the unadjusted coefficients, and age was a risk factor for the number of lost sextants. Senior men belonging to the category of older than 70 years increased their chance of having a higher number of lost sextants by 10%.

In relation to social capital, older people feeling unhappy had a greater chance of having a shallow periodontal pocket (43%), and of having a lost sextant (19%), when compared to those feeling happy or relatively happy. Elderly people unlikely to cooperate also had an 11% greater chance of having lost sextants.

Regarding the contextual variables, higher income inequality increased the chance of having lost sextants by 13%, and elderly individuals having a very high MHDI had a 66% lower chance of having shallow pockets. The FHS oral health coverage was a protective oral health factor in relation to gingival bleeding and dental calculus, and was a risk factor for the number of lost sextants. Fluoridation was a protective factor in relation to the number of lost sextants, as seen in Tables 3 and 4.

**Table 3:** Poisson multilevel modelling for gingival bleeding, calculus and shallow pocket SBSP15 (n = 2332).

			1-Gengival bleeding						2-Calculus						3-Shallow pocket					
Level Classification	Variables	Categories	RR^a^	IC95%		RR^b^	IC95%		RR^a^	IC95%		RR^b^	IC95%		RR^a^	IC95%		RR^b^	IC95%	
Individual factors	Gender	Male	Ref						Ref						Ref					
1 st level		Female	**0.74**	**0.67**	**0.80**	**0.76**	**0.69**	**0.83**	**0.72**	**0.66**	**0.78**	**0.72**	**0.66**	**0.79**	**0.75**	**0.66**	**0.84**	**0.74**	**0.65**	**0.84**
	Self-Skin color	White	Ref						Ref						Ref					
		Non-white	**1.26**	**1.15**	**1.39**	**1.26**	**1.14**	**1.39**	**1.25**	**1.15**	**1.35**	**1.21**	**1.10**	**1.33**	**1.26**	**1.10**	**1.43**	**1.25**	**1.10**	**1.43**
	Schooling	Continuous	**0.98**	**0.97**	**0.99**	0.98	0.97	1.00	**0.98**	**0.97**	**0.99**	0.98	0.97	0.99	0.99	0.97	1.02	—	—	—
	Age group	65–69	Ref						Ref						Ref					
		>70	1.05	0.96	1.15	1.03	0.9	1.14	**1.15**	**1.06**	**1.35**	1.16	1.06	1.26	1.00	0.9	1.13	—	—	—
	Income	≤1500	Ref						Ref						Ref					
		>1500	0.94	0.85	1.05	—	—	—	0.92	0.85	1.00	0.95	0.86	1.04	0.94	0.8	1.07	—	—	—
Social Capital	Cooperation	Very	Ref						Ref						Ref					
		Indifferent	**0.84**	**0.71**	**1**	**0.81**	**0.7**	**0.98**	0.94	0.82	1.08	—	—	—	**0.77**	**0.6**	**0.96**	**0.78**	**0.63**	**0.97**
		Improbably	0.99	0.85	1.16	1.01	0.9	1.19	1.05	0.92	1.18	—	—	—	0.83	0.7	1.01	**0.80**	**0.66**	**0.98**
	Security	Very	Ref						Ref						Ref					
		Indifferent	0.93	0.78	1.12	—	—	—	0.89	0.76	1.04	0.91	0.77	1.07	1.01	0.80	1.28	—	—	—
		Insecure	1.04	0.93	1.16	—	—	—	0.98	0.89	1.08	1.02	0.92	1.12	1.01	0.9	1.16	—	—	—
	Happiness	Very	Ref						Ref						Ref					
		indifferent	1.08	0.91	1.29	—	—	—	0.92	0.78	1.08	1.01	0.85	1.19	1.11	0.9	1.39	1.19	0.94	1.49
		Unhappiness	0.93	0.73	1.17	—	—	—	1.11	0.93	1.34	1.12	0.91	1.37	**1.37**	**1.1**	**1.77**	**1.43**	**1.10**	**1.86**
Contextual	Gini	Lower	Ref						Ref						Ref					
2nd Level		Middle	1.00	0.73	1.40	—	—	—	0.95	0.76	1.18	—	—	—	1.26	0.9	1.81	1.18	0.86	1.63
		Upper	1.19	0.88	1.63	—	—	—	1.03	0.84	1.27	—	—	—	1.05	0.7	1.49	1.05	0.75	1.46
	IDHM	<0,7	Ref						Ref						Ref					
		0,7–0,79	0.84	0.34	2.08	—	—	—	0.81	0.44	1.49	—	—	—	0.41	0.2	1.04	**0.44**	**0.19**	**0.99**
		>0,80	1.11	0.42	2.90	—	—	—	0.75	0.39	1.43	—	—	—	**0.32**	**0.1**	**0.86**	**0.44**	**0.18**	**1.00**
	FHS	<25%	Ref						Ref						Ref					
		25–50%	**0.62**	**0.42**	**0.91**	**0.66**	**0.4**	**0.99**	**0.78**	**0.61**	**0.99**	0.79	0.62	1	1.08	0.70	1.64	—	—	—
		> 50%	**0.67**	**0.47**	**0.96**	0.78	0.4	1.06	**0.79**	**0.62**	**0.99**	**0.75**	**0.60**	**0.95**	1.09	0.7	1.63	—	—	—
	fluoridation	0–70	Ref						Ref						Ref					
		70–90	0.80	0.30	2.13	—	—	—	1.19	0.65	2.19	1.02	0.54	1.92	1.14	0.40	3.21	—	—	—
		>90	0.81	0.56	1.19	—	—	—	0.85	0.66	1.09	0.85	0.67	1.08	0.86	0.6	1.31	—	—	—
	ICC null model	City	27.05(20.73–34.06)						19.11(17.63–25.05)						20.00(14.52–26.82)					

ICC–Intra Class Correlation

a–Unadjusted Odds Ratio

b–Adjusted Odds Ratio

Final model only variables (p<0.25)

**Table 4:** Poisson multilevel modelling for deep pocket and lost sextants SBSP15 (n = 2332).

			4-Deep pocket						5-Lost sextants					
Level	Variables	Categories	RR^a^	IC95%		RR^b^	IC95%		RR^a^	IC95%		RR^b^	IC95%	
	Gender	Male	Ref						Ref					
1 st level		Female	**0.62**	**0.49**	**0.78**	**0.65**	**0.51**	**0.84**	**1.10**	**1.05**	**1.16**	**1.10**	**1.04**	**1.16**
	Self-Skin color	White	Ref						Ref					
		Non-white	**2.10**	**1.64**	**2.69**	**2.14**	**1.63**	**2.81**	1.02	0.97	1.08	**—**	**—**	**—**
	Schooling	Continuous	0.97	0.94	1.00	0.98	0.95	1.01	**0.96**	**0.95**	**0.97**	**0.96**	**0.95**	**0.97**
	Age group	65–69	Ref						Ref					
		>70	0.83	0.65	1.05	0.82	0.63	1.07	**1.15**	**1.09**	**1.21**	**1.10**	**1.04**	**1.16**
	Income	≤1500	Ref						Ref					
		>1500	0.98	0.76	1.26	—	—	—	**0.91**	**0.86**	**0.96**	0.99	0.93	1.05
Social Capital	Cooperation	Very	Ref						Ref					
		Indifferent	1.03	0.71	1.50	—	—	—	0.96	0.87	1.04	0.94	0.85	1.03
		Improbably	0.94	0.65	1.36	—	—	—	**1.08**	**1.00**	**1.17**	**1.11**	**1.02**	**1.21**
	Security	Very	Ref						Ref					
		Indifferent	0.88	0.55	1.43	—	—	—	1.00	0.51	1.10	0.96	0.87	1.07
		Insecure	1.12	0.86	1.42	—	—	—	0.95	0.89	1.01	**0.92**	**0.86**	**0.99**
	Happiness	Very	Ref						Ref					
		Indifferent	1.15	0.73	1.81	—	—	—	**1.10**	**1.00**	**1.21**	**1.10**	**1.00**	**1.22**
		Unhappiness	0.91	0.52	1.58	—	—	—	**1.14**	**1.01**	**1.29**	**1.19**	**1.05**	**1.36**
Contextual	Gini	Lower	Ref						Ref					
2nd Level		Middle	0.75	0.39	1.40	—	—	—	1.10	0.98	1.24	**1.12**	**1.00**	**1.26**
		Upper	0.90	0.50	1.65	—	—	—	1.08	0.96	1.21	**1.13**	**1.01**	**1.27**
	IDHM	<0,7	Ref						Ref					
		0,7–0,79	1.13	0.18	6.94	—	—	—	0.92	0.65	1.31	—	—	—
		>0,80	0.53	0.07	3.75	—	—	—	0.98	0.67	1.41	—	—	—
	FHS	<25%	Ref						Ref					
		25–50%	0.80	0.39	1.63	—	—	—	1.10	0.95	1.27	**1.14**	**1.00**	**1.31**
		> 50%	0.97	0.49	1.91	—	—	—	1.07	0.93	1.22	**1.12**	**1.00**	**1.28**
	Fluoridation	0–70%	Ref						Ref					
		70%-90%	0.25	0.10	3.75	—	—	—	1.10	0.95	1.27	0.95	0.67	1.36
		>90%	1.52	0.67	3.40	—	—	—	0.91	0.79	1.05	**0.89**	**0.78**	**0.99**
	ICC null model	City	29.87(20.87–40.75)						17.83(12.32–25.09)					

ICC–Intra Class Correlation

a–Unadjusted Odds Ratio

b–Adjusted Odds Ratio

Final model only variables (p<0.25)

## Discussion

In our investigation, three important findings can be highlighted. First, social support variables were associated with periodontal diseases, which emphasize a holistic aspect of this oral condition prevention. Second, water fluoridation had a positive effect on preventing at least one lost sextant. Third, individual, contextual and social support variables had influences on periodontal outcomes in SBSP-15 survey.

The present investigation has some limitations and strengths. Because this was a cross-sectional study, causal direction cannot be established and only infers the association among the analyzed outcomes. Contextual variables could not be assessed because of individuals’ changes of address and of the long-term effect of fluoridation. About the social capital variables, only one question per domain was extracted from an integrated questionnaire SC-IQ[19], so these findings could be interpreted with caution. Further, the edentulous excluded people possibly have a link with the periodontal conditions. Strengths include the use of a representative sample and of recent data about the state of São Paulo (for the year 2015), which is the richest Brazilian state and the one with the largest gross domestic product (34.5%), representing approximately 645 Brazilian cities/towns.

The social support determined in the present research was based on analyzing the characteristics of cooperation, security and happiness[19] and emphasizes the holist component of periodontal disease(s) prevention. Social support can also express the trust that is established among groups, which favors organized collective action, and which can influence the development of the intelligentsia [20]. Individuals who have social networks may be better-informed, and, consequently, make better health choices [10]. In the present study, feeling unhappy was directly related to the clinical outcomes of periodontal disease, as a risk factor for shallow pockets and number of lost sextants. This emphasizes that the empowerment of elderly individuals in relation to their perception of life can influence and improve their situation, in terms of not only self-perception, but also the clinical outcome of periodontal diseases, which requires motivated collaboration to engage in daily brushing. In contrast, our findings showed that elders who are unlikely to be cooperative had a protective factor for bleeding and shallow pockets, and a risk factor for number of lost sextants. Senhem and Macke (2015)[11] explain that there are three types of social capital, and that they may represent different roles that networks can play in society. The explanation for cooperation has been a protective factor for the clinical outcome of shallow pockets refers to the fact that the perception of these individuals in relation to cooperation is restricted to family and closed groups (bonding social capital). At the same time, these individuals are unlikely to cooperate with other members, since they remain closed to "non-members" who want to join the group. These people do not cooperate with other members who are "outside the group" but, at the same time, easily develop cognitive dimensions and have strong ties with the health services[11]. This could explain the findings of the present study, since they may have connections with health services[11], and may encourage the inclusion guidelines for brushing and flossing. Future research needs to investigate the action of social capital against periodontal, dental and self-perceived treatment needs.

Socio Economic Status (SES) has already been related to periodontal outcomes[13,21,22,23,24,25,26,27] supporting the findings that individual predisposing and individual enabling variables were associated with periodontal diseases[28], denoting periodontal health inequalities.

Fluoridation of the public water supply has been associated with higher functional dentition in Brazilian adults [29], and with a decrease in prevalence of dental caries in Brazilian population, a major determinant of future dental losses[30]. In our study, water fluoridation was associated with the number of lost sextants (protective factor), and it is important for municipal public policies[30], may influence pre-existing social inequalities and perhaps tooth loss and protein intake[31].

Context variables such as Gini coefficient and MHDI are also worth considering, because they influence the outcomes of periodontal disease significantly. Greater inequality in the distribution of income was a risk factor for the number of lost sextants, and a very high MHDI was a protection factor for clinical outcomes of shallow pockets, thus evidencing the relation of contextual factors to periodontal diseases of elderly individuals, corroborating other findings[13,28].

CPITN has some limitations, since it is based on partial recordings of probing depth, instead of full-mouth recordings of attachment loss. Consequently, there is evidence that CPITN underestimates the more severe periodontal conditions in terms of both prevalence and severity, overestimates prevalence and severity of periodontal disease in younger subjects and underestimates such parameters in elderly populations [32]. CPITN is used in nationwide (SB Brasil) and regional (SBSP-15) surveys in Brazil, which is useful for comparison with other countries.

According to Papapanou (2017)[33], periodontitis should no longer be defined solely by gingival/periodontal inflammation in the presence of increased probing depths and clinical attachment loss, but should incorporate additional dimensions capturing impaired function, esthetics, and impact on general health and quality of life. This multidimensional approach with social support variables, is a first step to understand that feeling happy could improve periodontal status and oral health related quality of life of Elders [34].

FHS coverage was positively associated with the clinical characteristics of periodontal disease, such as the presence of gingival bleeding and periodontal calculus, corroborating another finding [13]. Primary health care teams have provided guidance ever since the FHS reorganized primary health care in Brazil. Dental surgeons in FHS- Oral Health Team units perform health prevention and promotion activities, such as oral hygiene guidelines for elderly groups, as well as clinical treatment, such as dental calculus scraping, routinely. For the treatment of more advanced periodontal disease, such as deep pockets, elderly patients must be referred to the Dental Specialties Centers (DSCs—secondary care), which may help FHS coverage exert a positive effect on oral healthcare outcomes.

## Conclusions

The study showed evidence that individual (gender and self-perceived skin color), contextual (coverage of the family health strategy and water fluoridation) and social support factors (feeling unhappy) are associated with the clinical outcomes of periodontal diseases in Brazilian elders. This reinforces the need for transdisciplinary actions in the Family Health Strategy stimulating work together and inter sectoral collaboration between FHS and NASF (Family Health Support Center).
